# Sarcomatoid chromophobe renal cell carcinoma: Cytohistopathological correlation of a case

**DOI:** 10.4103/0970-9371.66690

**Published:** 2010-01

**Authors:** Indranil Chakrabarti, Amita Giri, Kaushik Majumdar, DE Anuradha

**Affiliations:** Department of Pathology, North Bengal Medical College, Sushrutanagar, Darjeeling, India

**Keywords:** Cytology, kidney, pleomorphic sarcoma, sarcomatoid chromophobe renal cell carcinoma

## Abstract

Sarcomatoid renal cell carcinomas of the kidney are rare neoplasms constituting about 1-5% of all renal malignant neoplasms. These are aggressive tumors and are commonly associated with conventional (clear cell) renal cell carcinomas, but cases associated with chromophobe renal cell carcinomas are sparse. Cytological features of such lesions have rarely been reported. Here, we report a unique case of a 48-year-old male patient who presented with right flank lump and pain. A fine needle aspiration was performed from the lesion under ultrasound guidance and a cytological diagnosis of pleomorphic sarcoma was made. A right-sided radical nephrectomy was carried out and subsequent histopathology revealed a sarcomatoid renal cell carcinoma with wide areas of necrosis coexisting with chromophobe renal cell carcinoma with calcification. Differentiation of pleomorphic sarcoma from a sarcomatoid renal cell carcinoma is, thus, challenging from cytopathology smears and the differential diagnoses should always be borne in mind while giving a cytopathological opinion.

## Introduction

Sarcomatoid renal cell carcinomas of the kidney are rare neoplasms constituting about 1-5% of all renal malignant neoplasms. These are clinically aggressive tumors and are more commonly associated with conventional (clear cell) renal cell carcinomas, but very few cases have been reported so far, in which they have been associated with chromophobe renal cell carcinomas, collecting duct carcinomas and papillary carcinomas. The paradox lies in the fact that although chromophobe renal cell carcinomas have traditionally been associated with a more favorable prognosis than clear cell carcinomas, sarcomatoid carcinomas signify a poor prognosis.[1] Some authors, however, opine that chromophobe renal cell carcinomas among all renal cell carcinomas are more likely to have sarcomatoid transformation.[2] Only very few cases have been reported in the literature and diagnoses on cytological aspirates are sparse.[3]

## Case Report

A 48-year-old male patient presented with pain and progressively increasing swelling of six months duration in the right hypochondrium. Ultrasonography revealed a large nine cm swelling in the upper pole of the right kidney. No other abdominal pathology was detected on ultrasonography. Ultrasound-guided fine needle aspiration (FNAC) was carried out. Multiple passes were performed with a 24 gauge needle fitted to a 10 ml syringe and the smears were stained with hematoxylin and eosin stain and May-Grünwald – Giemsa stain.

**Figure 1:**
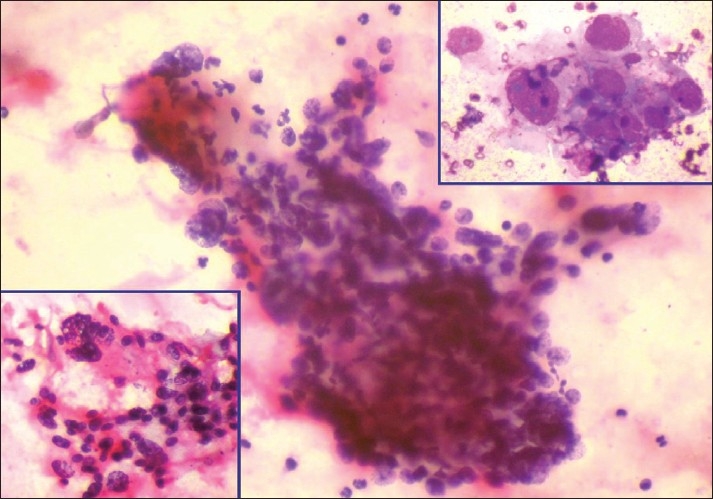
Microphotograph from cytology smears showing clusters of round to oval malignant cells with nuclear pleomorphism, overlapping, focal spindling and bizarre cells (H and E, ×400). Lower left inset shows another cluster of pleomorphic malignant cells (H and E, ×400). Upper right inset shows bizarre multinucleated tumor giant cells (MGG, ×1000)

The cellular smears showed presence of clusters of round to oval, pleomorphic, malignant cells along with some bizarre spindle cell clusters. Tumor giant cells were present along with tumor diathesis. The background was mostly hemorrhagic [Figure 1]. A cytopathological diagnosis of a pleomorphic sarcoma was made.

**Figure 2:**
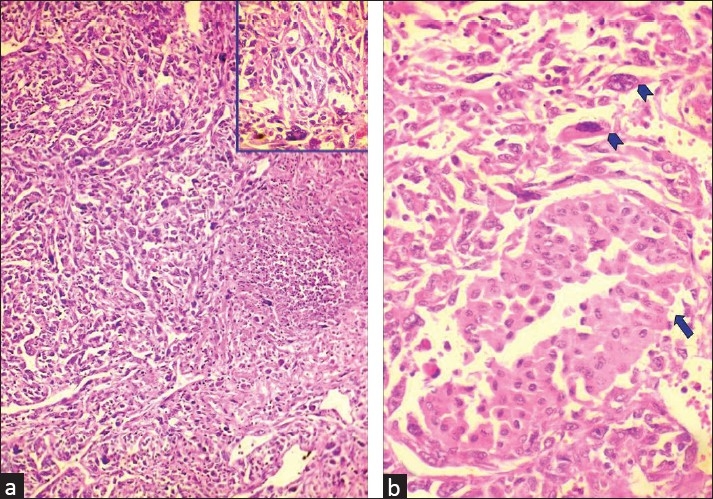
(a) Microphotograph showing sarcomatoid renal cell carcinoma with bizarre tumor giant cells and necrosis (H and E, ×100). Inset showing tumor giant cell among pleomorphic cells (H and E, ×400).(b) Microphotograph showing chromophobe renal cell carcinoma (arrow) admixed with sarcomatoid areas with bizarre cells and giant cells (arrowheads) (H and E, ×400)

A right-sided radical nephrectomy was performed and the tissue was submitted for histopathology. Gross examination showed a 6.5 cm × 6.0 cm swelling in the upper pole of the right kidney arising from the medulla, with extension to the renal capsule. Few irregular grayish tissue bits, altogether 4.0 cm × 3.0 cm, were also sent along. No lymph nodes were resected. The cut-section was mostly firm and grayish-white in color, with foci of hemorrhage and necrosis. Sections were given from multiple areas. Microscopic examination revealed sarcomatoid renal cell carcinoma with presence of bizarre tumor giant cells and wide areas of necrosis [Figure 2a].

Areas of sarcomatoid renal cell carcinoma coexisting with chromophobe renal cell carcinoma were identified [Figure 2b].

Foci of calcification were noted in the chromophobe areas. The malignant cells were seen to invade the renal capsule. A final histopathological diagnosis of sarcomatoid renal cell carcinoma coexisting with chromophobe renal cell carcinoma was made.

## Discussion

Sarcomatoid renal cell carcinomas of thekidney, constituting about 1-5% of all renal malignant neoplasms,[1] are clinically aggressive tumors with rapid spread and poor overall survival. Some studies have shown that sarcomatoid renal cell carcinomas are commonly associated with conventional (clear cell) renal cell carcinomas[1] while some other studies show that they are more likely to be associated with chromophobe renal cell carcinomas.[2] There are only very few cases reported in the literature of sarcomatoid chromophobe renal cell carcinoma.[4,5] More so, reports on diagnosis based on cytological aspirates are sparse. Auger *et al*.[6] reported 15 cases of sarcomatoid renal cell carcinoma (nine primary tumors and six metastases) diagnosed by FNAC, of which 12 cases showed clear cell or granular cell renal cell carcinoma associated with sarcomatoid component. Gadre *et al*.[3] reported a case of sarcomatoid renal cell carcinoma with unusual coexpression of the S-100 protein, based on cytology. Yong *et al*.[7] described an interesting case of sarcomatoid renal cell carcinoma metastatic to the breast. In the present case, even on multiple aspirations, the smears yielded cells resembling a sarcoma, and it was not until a thorough histopathological sampling that the chromophobe areas with sarcomatoid renal cell carcinoma were identified. Differentiation of pleomorphic sarcoma from a sarcomatoid renal cell carcinoma is, thus, challenging from cytopathology smears and the differential diagnoses should always be borne in mind while giving a cytopathological opinion.

Atypical epithelioid angiomyolipoma,[8] although extremely rare, is another important differential diagnosis on aspiration cytology.

Detection of sarcomatoid renal cell carcinoma has an important prognostic significance, as reflected by its aggressive behavior, propensity to metastasize and association with the overall poor survival. Fortunately, the patient in this case presented early with no detectable metastasis. In case of metastatic renal cell carcinoma, now-a-days, various treatment modalities like immunotherapy (with interferon-alpha, interleukin-2) and targeted therapy with drugs having anti-angiogenic effects (Sorafenib, Sunitinib, temsirolimus, etc.)[9] have met with encouraging success. The prognosis of pleomorphic renal sarcomas, generally treated with chemotherapy, however, continues to be poor. The accurate diagnosis of such tumors, thus, is of paramount importance. In this context, although a needle core biopsy usually provides more information and is a preferred method, FNAC proves to be a rapid, inexpensive, less-invasive as well as fairly accurate tool in the early detection of these types of tumors. However, histopathology with thorough sampling remains the gold standard for confirmation of the diagnosis.

## References

[1] Cohen RJ, McNeal JE, Susman M, Sellner LN, Iacopetta BJ, Weinstein SL (2000). Sarcomatoid renal cell carcinoma of papillary origin: A case report and cytogenic evaluation. Arch Pathol Lab Med.

[2] Akhtar M, Tulbah A, Kardar AH, Ali MA (1997). Sarcomatoid renal cell carcinoma: The chromophobe connection. Am J Surg Pathol.

[3] Gadre SA, Math SK, Elfeel KA, Farghaly H (2009). Cytology of a sarcomatoid renal cell carcinoma with unusual coexpression of S-100 protein: A case report, review of the literature and cytologic-histologic correlation. Diagn Cytopathol.

[4] Parada D, Peña K, Moreira O (2006). Sarcomatoid chromophobe renal cell carcinoma: A case report and review of the literature. Arch Esp Urol.

[5] Abrahams NA, Ayala AG, Czerniak B (2003). Chromophobe renal cell carcinoma with sarcomatoid transformation. Ann Diagn Pathol.

[6] Auger M, Katz RL, Sella A, Ordóñez NG, Lawrence DD, Ro JY (1993). Fine-needle aspiration cytology of sarcomatoid renal cell carcinoma: A morphologic and immunocytochemical study of 15 cases. Diagn Cytopathol.

[7] Ding GT, Hwang JS, Tan PH (2007). Sarcomatoid renal cell carcinoma metastatic to the breast: Report of a case with diagnosis on fine needle aspiration cytology. Acta Cytol.

[8] Delgado R, de Leon Bojorge B, Albores-Saavedra J (1998). Atypical angiomyolipoma of the kidney: A distinct morphologic variant that is easily confused with a variety of malignant neoplasms. Cancer.

[9] Molina AM, Motzer RJ (2008). Current algorithms and prognostic factors in the treatment of metastatic renal cell carcinoma. Clin Genitourin Cancer.

